# Normal and Dysregulated Sphingolipid Metabolism: Contributions to Podocyte Injury and Beyond

**DOI:** 10.3390/cells13110890

**Published:** 2024-05-22

**Authors:** Matthew Tolerico, Sandra Merscher, Alessia Fornoni

**Affiliations:** Peggy and Harold Katz Family Drug Discovery Center, Miller School of Medicine, University of Miami, Miami, FL 33136, USA; mxt1001@miami.edu

**Keywords:** podocyte, sphingolipid, glomerular disease, ganglioside, cerebroside, podocytopathies, ceramide, sphingosine-1-phosphate, lipid rafts

## Abstract

Podocyte health is vital for maintaining proper glomerular filtration in the kidney. Interdigitating foot processes from podocytes form slit diaphragms which regulate the filtration of molecules through size and charge selectivity. The abundance of lipid rafts, which are ordered membrane domains rich in cholesterol and sphingolipids, near the slit diaphragm highlights the importance of lipid metabolism in podocyte health. Emerging research shows the importance of sphingolipid metabolism to podocyte health through structural and signaling roles. Dysregulation in sphingolipid metabolism has been shown to cause podocyte injury and drive glomerular disease progression. In this review, we discuss the structure and metabolism of sphingolipids, as well as their role in proper podocyte function and how alterations in sphingolipid metabolism contributes to podocyte injury and drives glomerular disease progression.

## 1. Introduction

Podocytes are terminally differentiated epithelial cells that serve an essential role in the filtration capability of the kidney. Foot processes that extend off podocytes form an interdigitating pattern with foot processes from neighboring podocytes, leaving in between them filtration slits, called the slit diaphragm. The slit diaphragm is composed of specialized slit diaphragm proteins, such as nephrin and podocin, which allow for the filtration of small molecules [1]. The loss of function mutations in slit diaphragm proteins leads to podocyte damage and improper filtration, resulting in proteinuria and nephrotic syndrome [2]. Many of these slit diaphragm proteins have been found to localize to sphingolipid-rich lipid rafts [3,4]. Sphingolipids comprise a diverse family of lipids first identified by Johann Ludwig Wilhelm Thudichum in 1879 [5]. Due to their enigmatic nature, the first identified sphingolipid “sphingosine” was named after the Sphinx. Although initially thought to simply provide a structural role in eukaryote cell membranes, recent discoveries highlight a role for sphingolipids that encompasses a wide range of bioactive functions [6]. Furthermore, recent research has highlighted the importance of sphingolipid metabolism in podocyte health and the pathogenesis of nephrotic syndrome. 

## 2. Overview of Sphingolipids

**Figure 1:**
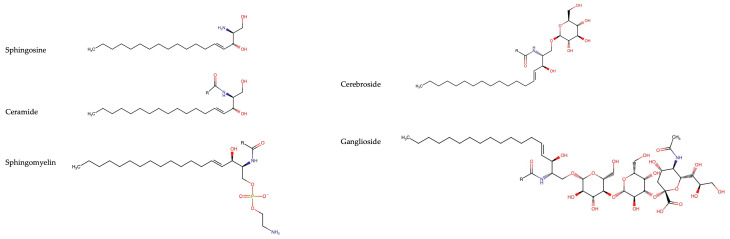
Overview of typical sphingolipid structures. Sphingosine is an 18-carbon amino alcohol with a double bond between carbons 4 and 5 and comprises the backbone of all sphingolipids. Ceramides are a class of sphingolipids in which a sphingosine is attached to a fatty acid (R) of varying lengths through an amide linkage. Sphingomyelins consist of a ceramide molecule bound to a phosphocholine group at the primary carbon. Cerebrosides are a class of glycosphingolipids in which a ceramide is bound at the primary carbon to either glucose or galactose. The cerebroside shown here is glucocerebroside. Gangliosides consist of a ceramide bound to one or more sugar moieties that contain at least one sialic acid linked to the sugar chain. The ganglioside shown is GM3, which consists of a ceramide linked to a glucose which is attached to a galactose. The galactose has the sialic acid, *N*-acetylneuraminic acid or NANA, linked to it.

The major classes of sphingolipids are: sphingoid bases, ceramides, sphingomyelins, and glycosphingolipids (cerebrosides and gangliosides). Due to the large number of possible headgroups, sphingoid bases, and fatty acid tail lengths, the number of possible sphingolipid species is in the tens of thousands, making it the most structurally diverse class of lipids [7]. Sphingolipids are amphipathic due to their hydrophobic hydrocarbon chains and their hydrophilic polar head groups. This property allows them to localize in phospholipid membranes, in particular, cholesterol-rich lipid rafts, with their hydrocarbons embedded in the membrane and their polar head groups facing towards the cytosol or extracellular space [8]. The typical structures of sphingolipids are shown in Figure 1. 

### 2.1. Sphingoid Bases

Sphingoid bases are aliphatic amino alcohols that comprise the backbone of all sphingolipids; however, sphingoid bases exist on their own as well and provide important roles in cell signaling and survival [9]. Over one hundred different sphingoid bases have been identified, but these are often unique to a specific cell type [10]. The most abundant sphingoid base in eukaryotic cells is sphingosine ((2S,3R,4E)-2-amino-4-octadecene-1,3-diol) [11]. Sphingosine is an eighteen-carbon aliphatic chain, with hydroxyl groups attached to carbons 1 and 3, an amine group attached to position 2, and a *trans*-double bond in position 4. Sphingosine serves as the backbones for more complex sphingolipids which will be discussed later. Dihydrosphingosine or sphinganine is another common sphingoid base found in eukaryotic cells. The structure of sphinganine is identical to sphingosine but lacks the double bond at position 4. Phytosphingosine is an abundant sphingoid base that has an additional hydroxyl group at position 4. Phytosphingosine comprise the backbone of many sphingolipids in plants; however, it has also been identified in various eukaryotic cell types [12]. The primary hydroxyl group of sphingosines can be phosphorylated to form the bioactive metabolite sphingosine-1-phosphate (S1P). S1P can be exported to the extracellular space where it can activate one of its five identified G-protein-coupled receptors, termed sphingosine-1-phosphate receptor 1-5 (S1PR1-5). S1P has been an increasingly researched sphingolipid due to its role in the pathogenesis of a variety of diseases including cancers, inflammation, and neurological disorders [13]. 

### 2.2. Ceramides

Ceramides are simple sphingolipids whose structure is composed of a sphingoid base with an amide linked fatty acid and a primary terminal hydroxyl group. The length of the fatty acid tail generally ranges from 14 to 26 carbons long, with most of them being saturated or mono-unsaturated [14]. Ceramides serve as the centerpiece of sphingolipid metabolism as more complex sphingolipids are generated from ceramide. Due to the presence of predominantly saturated fatty acids in ceramides, ceramides can form raft-like structures [15]. Generally, ceramides are thought to induce apoptosis and inhibit cellular proliferation, but the exact mechanisms are not fully understood [16]. Like sphingosine, ceramides can be phosphorylated at the primary hydroxyl group to generate ceramide-1-phosphate (C1P). C1P appears to have an antagonistic function compared to ceramides, and C1P is most often considered to have anti-apoptotic and pro-proliferative functions [17]. However, the effects of ceramides and C1P depend heavily on the fatty acid chain lengths and cell type where the lipids are found. 

### 2.3. Sphingomyelins

Sphingomyelins are complex sphingolipids which are composed of a ceramide linked by a phosphodiester bond to phosphocholine. Sphingomyelins linked to phosphoethanolamine exist in high concentrations in invertebrates [18]. Sphingomyelin is the most abundant sphingolipid in animals, representing 85% of all sphingolipids, and is structurally vital for cell membranes. Sphingomyelins make up 20% of plasma membrane lipids and are concentrated in lipid rafts on the exoplasmic leaflet [19]. Sphingomyelin is also highly abundant in the myelin sheath surrounding nerve cell axons, where it provides an insulating environment, and serves as a reservoir to produce ceramides at membranes, which then can be converted to more complex sphingolipids or sphingosine. 

### 2.4. Cerebrosides

Cerebrosides are one type of glycosphingolipid, a ceramide linked to sugar molecules, in which one sugar moiety, either glucose or galactose, is attached to ceramide at the terminal hydroxyl group by a glycosidic bond. The two types of cerebrosides are glucocerebrosides (glucosylceramides) and galactocerebrosides (galactosylceramides) in which glucose or galactose is linked to ceramide respectfully. Galactosylceramide is a major component of brain tissue, hence the name “cerebroside”. Galactosylceramides compose around 2% of the dry weight of grey matter and 12% of white matter. Moreover, galactosylceramides make up around 23% of myelin lipids where they insulate axons of neurons and provide structure to the extended membranes of oligodendrocytes [20]. Glucocerebrosides are concentrated in the skin where they are essential for maintaining the water permeability of the barrier [21]. Cerebrosides can form large amounts of hydrogen-bonding interactions between the polar hydrogens of the sugar and the hydroxy and amide groups of the sphingosine bases, resulting in compact packing [22]. Like other sphingolipids, cerebrosides are found concentrated in “lipid rafts”. 

### 2.5. Gangliosides

Gangliosides are the most complex class of sphingolipids and named due to their abundance in ganglion cells. They are composed of a ceramide attached to an oligosaccharide chain with one or more sialic acids linked to the sugar chain. Gangliosides can differ from one another depending on the ceramide structure (sphingoid base and fatty acid tail), the number and type of sugar chain (glucose, galactose, etc.), and the number, location, and type of sialic acid. Paralleling their complex structure, gangliosides are also unique in that they are only found in vertebrates [23]. Like sphingomyelin and cerebrosides, gangliosides are found concentrated in neural tissue. Gangliosides are enriched in the brain where they comprise 80% of all glycans and 75% of all sialic acids in neuronal membranes [24]. They concentrate in the outer leaflet of the cell membrane within lipid rafts. Due to the high polarity of the saccharide chain and the high hydrophobicity of the ceramide tail, gangliosides are embedded in the outer leaflet with the sugar chain facing towards the extracellular space [25]. Gangliosides play an important role in regulating cell signaling through a variety of mechanisms. The sugar chain on gangliosides can bind to the sugar chains on membrane proteins or by binding directly to amino acids [25]. They can also bind directly to Siglec proteins, which are sialic-acid-binding lectins found on immune cells and the myelin sheath [26]. Ganglioside have been shown to modulate the function of a variety of ligands such as interferon, insulin, and growth factors by either directly binding to the ligand or its receptor [27]. Gangliosides are also responsible for the entry of numerous bacterial toxins into cells such as cholera and botulism [28]. They are also important for host recognition by the immune system. Guillain–Barré syndrome is a neuromuscular syndrome in which the body produces antibodies for various gangliosides. This results in the immune system attacking the myelin sheath on peripheral nerves, leading to muscle weakness, breathing issues, and possible death [29]. Tay–Sachs is a recessive genetic disorder that has a very high incidence in Ashkenazi Jews (1 in 30 are carriers). Tay–Sachs is caused by a mutation in the *HEXA* gene resulting in a dysfunctional hexosaminidase A. Hexosaminidase A is responsible for degrading the ganglioside GM2 to GM3. This accumulation of GM2 leads to the destruction and improper function of nervous tissue in the brain and spinal cord, usually resulting in death before the age of five in those affected [30]. 

## 3. Sphingolipid Metabolism

**Figure 2:**
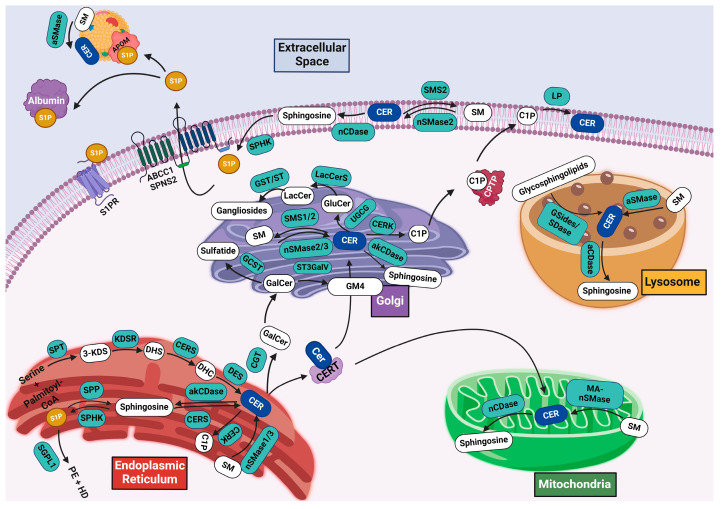
Overview of sphingolipid metabolism. ABCC1, ATP-binding cassette transporter C1; aCDase, acid ceramidase; akCDase, alkaline ceramidase; APOM, apolipoprotein M; aSMase, acid sphingomyelinase; CER, ceramide; CERK, ceramide kinase; CERS, ceramide synthase; CGT, Ceramide galactosyltransferase; CPTP, C1P transport protein; C1P, ceramide-1-phosphate; DES, dihydroceramide desaturase; DHC, dihydroceramide; DHS, Dihydrosphingosine; GluCer, glucosylceramide; GCST, galactosylceramide sulfotransferase; GSides, glycosides; GST, glycosyltransferase; HD, hexadecenal; 3-KDS, 3-ketodihydrosphinganine; KDSR, 3-ketodihydrosphingosine reductase; LacCer, lactosylceramide; LacCerS, LacCer synthase; LP, lipid phosphatase; MA-nSMase, mitochondria-associated neutral sphingomyelinase; nCDase, neutral ceramidase; nSMase, neutral sphingomyelinase; PE, phosphoethanolamine; SDase, sialidase; SGPL1, sphingosine-1-phosphate lyase 1; SM, sphingomyelin; SMS, sphingomyelin synthase; SPHK, sphingosine kinase; Spns2, sphingolipid transporter 2; SPP, sphingosine-1-phosphate phosphatase; SPT, serine-palmitoyl-transferase; ST, siayltransferases; ST3GalV, siayltransferases 5; S1P, sphingosine-1-phosphate; S1PR, sphingosine-1-phosphate receptor; and UGCG, UDP-glucose ceramide glucosyltransferase.

The generation of sphingolipids is a tightly regulated process controlled by key enzymes in specific cellular compartments. The key pathways in sphingolipid metabolism are (1) de novo synthesis, where sphingolipids are generated from non-sphingolipid precursors, (2) the salvage pathway, in which complex sphingolipids are broken down and recycled back into ceramide, (3) ceramide catabolism, which converts ceramides to sphingosine and bioactive sphingolipids, and (4) the generation of complex sphingolipids. An overview of sphingolipid metabolism is shown in Figure 2. 

### 3.1. De Novo Synthesis

De novo sphingolipid synthesis begins in the cytosolic leaflet of the endoplasmic reticulum where serine and fatty-acyl coenzyme A are condensed by the rate-limiting enzyme serine palmitoyltransferase (SPT) to generate 3-ketosphingane (3-ketodihydrosphingosine). Palmitoyl-CoA is the predominantly used fatty acyl-CoA in this process; however, the preferential substrate is determined by the subunit composition of the SPT enzyme [31]. This reaction is regulated by orosomucoid-like proteins and small subunit SPTs which inhibit or facilitate the activity of SPT [32,33]. Mutations in subunits of SPT can result in the utilization of the amino acids glycine and alanine instead of serine, resulting in “dead end” ceramides which cannot be further modified due to the lack of a hydroxyl group on the primary carbon [34]. 

The C3 ketone of 3-ketosphinganine is then reduced by 3-ketodihydrosphingosine reductase (KDSR) in an NADPH-dependent mechanism to produce sphinganine (dihydrosphingosine). Sphinganine can be converted to either sphinganine-1-phosphate by sphingosine kinase through ATP-dependent phosphorylation, to phytosingosine by dihydrosphingosine hydroxylase, or, most commonly, to dihydroceramide by ceramide synthase (CerS) through the amide linkage of an acyl group from a fatty acyl-CoA to sphinganine. Six different ceramide synthases have been identified in mammals with varying degrees of preference for acyl substrate usage. Ceramide synthase 1 primarily uses C18-CoA, CerS2 prefers C22-23-CoAs, CerS3 prefers C26-CoA or longer acyls, Cer4S uses C18-C22-CoAs, and CerS5 and CerS6 mainly use C-16-CoA [35]. The ability to produce varying lengths of ceramides allow for their modulation based on cellular needs as well as tissue specificity. De novo synthesis is finally concluded by the addition of a double bond to dihydroceramide by dihydroceramide desaturase (DES) to generate ceramide. Ceramide will then be transported to the Golgi apparatus or the mitochondria by the soluble ceramide transfer protein (CERT) where it can be utilized to generate further sphingolipid species [36]. 

### 3.2. Salvage Pathway

Ceramide can also be generated through the breakdown and recycling of complex sphingolipids such as sphingomyelin and glycosphingolipids at the plasma membrane, at the Golgi apparatus, in lysosomes, or in mitochondria. Sphingomyelinases (SMases) hydrolyze sphingomyelin to ceramide and phosphocholine. Three classes of SMases have been identified based on their preferential pH: acid SMases, neutral SMases, and alkaline SMases. 

Acid SMases are directed to either the lysosomal or secretory pathway depending on mannose-6-phosphate residue presence. Acid SMases that contain mannose-6-phosphate residues are trafficked through the lysosomal pathway to endolysosomes where it degrades sphingomyelin [37]. Acid SMases that are not mannose-6-phosphorylated enter the Golgi secretory pathway where they are trafficked to the extracellular space [11]. Secretory acid SMases can break down sphingomyelin that is located on the outer leaflet of the cellular membrane as well as sphingomyelin that is located on lipoproteins in the plasma [38]. Four different neutral SMases (nSMases) have been identified, nSMase 1–3 and mitochondrial-associated nSMase (MA-nSMase). nSMase 1 is located in the endoplasmic reticulum, nSMase 2 in the plasma membrane and the Golgi apparatus, nSMase 3 in the endoplasmic reticulum and Golgi apparatus, and MA-nSMase in mitochondria. nSMase 3 is largely specific to skeletal and muscle tissue, while alkaline SMases are unique to the liver and intestine where they function to degrade dietary sphingomyelin. Interestingly, they have very little homology to the acid and neutral SMases [39]. MA-nSMase has been shown to induce apoptosis through ceramide accumulation in the mitochondria. 

Glycosphingolipids are degraded to ceramide in lysosomes. GM1 is degraded to GM2 through the cleavage of the terminal β-D-galactose by the enzyme GM1-β-galactosidase [40]. The terminal N-acetylgalctosamine from GM2 is then removed by hexosaminidase A (HexA) to generate GM3. HexA is also responsible for degraded globosides. GM3 is hydrolyzed by sialidase to remove the sialic acid residues to produce LacCer, which is then hydrolyzed to GlcCer by GM1-β-galactosidase or galactosylceramide-β-galactosidase. Finally, GlcCer are hydrolyzed by glucosylceramide-β-glucosidase to generate ceramides [41]. 

### 3.3. Ceramide Catabolism and Bioactive Sphingolipids

Ceramide is deacetylated by a group of enzymes called ceramidases into sphingosine and free fatty acyl-CoA. Similarly to SMases, ceramidases are grouped based on their preferential pH: acid, neutral, and alkaline. Acid ceramidase is in the lysosomes and has a higher activity for ceramides of small and medium lengths C8 to C14. Neutral ceramidase is found at the plasma membrane and in mitochondria, where it acts on ceramides with a preference for the C16 and C18 length. Alkaline ceramidases are in the ER and Golgi apparatus and preferentially degrade longer chain ceramides C20–C24 [42]. 

Ceramide can also be phosphorylated into ceramide-1-phosphate by ceramide kinase in the ER and the Golgi. Ceramide-1-phosphate is then transported to the plasma membrane by ceramide phosphate transfer protein (CPTP) [43]. C1P can be dephosphorylated to ceramide at the cell membrane by ceramide phosphatase, lipid phosphatases, and sphingomyelin phosphodiesterase acid-like 3b [44,45]. 

Sphingosine can be phosphorylated in an ATP-dependent mechanism at the C-1 hydroxyl group by two sphingosine kinases, sphingosine kinase 1 (SK1) and sphingosine kinase 2 (SK2), into sphingosine-1-phosphate (S1P). Both SKs are cytosolic enzymes but can associate with membranes. SK1 can translocate from the cytoplasm to the plasma membrane due to phosphorylation by ERK2 [46]. This translocation leads to an increase in S1P production and release into the extracellular space. The phosphorylation of SK1 by ERK2 has been shown to be necessary for many different growth factors [47,48,49,50]. SK1 has also been identified in the nucleus. However, the role and mechanism of SK1 in the nucleus is unclear [51]. SK1 has also been shown to be secreted into the extracellular space where it can generate S1P on the outer leaflet. However, the importance of extracellular SK1 has not been identified [52].

Less is known about SK2. It is largely localized to the nucleus and perinuclear regions. Evidence suggests it may play a role in apoptosis regulation as overexpression leads to increased apoptosis [53]. SK2 also localizes with sphingosine-1-phosphate phosphatase which dephosphorylates S1P to sphingosine, suggesting it may play a role in ceramide recycling [54]. 

S1P is exported to the cytosol by the transport proteins spinster homologue 2 (SPNS2) and ATP-binding cassette (ABC) transporters [55,56]. In the extracellular space, S1P binds to its chaperone proteins either albumin or apolipoprotein M, which is found on high-density lipoprotein particles. S1P is then transported through the circulation to target cells where it can bind to one of its five G-protein-coupled receptors, termed S1P receptor 1–5, and activate downstream pathways [57]. 

The final step in sphingolipid catabolism is the degradation of S1P by S1P lyase (SPL) to hexadecenal and phosphoethanolamine. SPL is a type-I transmembrane protein exclusively localized to the ER with its catalytic site facing towards the cytosol allowing it to interact with cytosolic S1P [58]. Therefore, sphingolipid metabolism begins in the ER with de novo synthesis and concludes in the ER with S1P lyase. 

### 3.4. Synthesis of Complex Sphingolipids

Following the formation of ceramide through either de novo synthesis or the salvage pathway, ceramide can be utilized to generate more complex sphingolipids such as sphingomyelin and glycosphingolipids. 

#### 3.4.1. Sphingomyelin Synthesis

Ceramide generated in the ER is transferred to the inner leaflet of the Golgi apparatus by ceramide transport protein (CERT) [59]. A sphingomyelin synthase (SMS) catalyzes the transfer of a phosphocholine group from phosphatidylcholine to ceramide, thus forming sphingomyelin and diacylglycerol (DAG). There are three sphingomyelin synthases, SMS1, SMS2, and sphingomyelin-related synthase protein (SMSr). SMS1 and SMS2 have a similar homology and function, with both generating sphingomyelin (ceramide phosphocholine) utilizing phosphatidylcholine in the Golgi apparatus; however, SMS2 is also localized to the plasma membrane [60]. SMSr is unique in that it is localized exclusively to the trans-ER and forms sphingomyelin (ceramide phosphoethanolamine) from transferring a phosphoethanolamine from phosphatidylethanolamine to ceramide [61]. 

#### 3.4.2. Glycosphingolipid Synthesis

The formation of one of the cerebrosides, galactosylceramides (GalCer), occurs in the ER. Ceramide galactosyltransferase (CGT) is localized to the ER with its catalytic site facing towards the ER lumen. CGT catalyzes the galactosylation of ceramide utilizing uridine diphosphate galactose (UDP-Gal) as a substrate [62]. GalCer can then be transported by vesicles to the Golgi apparatus for further modification. Galactosylceramide sulfotransferase located in the Golgi apparatus catalyzes the formation of sulfatides through the transfer of a sulfate group from 3′-phosphoadenosine-5′-phosphosulfate (PAPS) to the galactose moiety on galactosylceramides to generate adenosine 3′,5′-bisphosphate and a galactosylceramidesulfate (sulfatide) [39]. The simple ganglioside Neu5Acα2-3GalβCer (GM4) can also be formed from GalCer. Siayltransferase ST3GalV catalyzes the transfer of a sialyl group (N-acetyl-alpha-neuraminyl or NeuAc) from CMP-NeuAc (cytidine 5’-monophosphate-NeuAc) to GalCer, forming GM4 in the Golgi apparatus [63]. 

The formation of glucosylceramide (GlcCer) occurs in the *cis*-Golgi network where UDP-glucose ceramide glucosyltransferase (UGCG) transfers a glucose residue from UDP-glucose to the C1 hydroxyl group of a ceramide [64]. β-1,4-galactosyltransferases (LacCer synthases) drive the formation of lactosylceramide (LacCer) by the addition of a galactose to GlcCer. LacCer can then interact with more glycosyltransferases and siayltransferases to form more complex globosides and gangliosides [65].

## 4. Sphingolipids in Podocytes

Research has highlighted the importance of lipid rafts in maintaining the proper slit diaphragm structure and function in podocytes [66,67,68]. Therefore, the proper composition of sphingolipids in lipid rafts is essential for the maintenance of proper filtration. Complex sphingolipids have been shown to interact with proteins in podocyte lipid rafts and modulate their effects. For example, Nephrin, a podocyte-specific slit diaphragm protein, associates with 9-O-acetylated GD3, and antibodies for 9-O-acetylated GD3 cause foot process effacement and slit diagram morphological changes [3]. Furthermore, it has been suggested that many sphingolipid species regulate the charge-selective filtration barrier of the slit diaphragm due to their abundance and charge [67]. The ganglioside GM3 seems to play an important role in the regulation of the actin cytoskeleton of podocytes. It was previously shown that GM3 binds to soluble vascular endothelial growth factor receptor 1 (Flt1) at the foot processes and is essential for foot process health. The deletion of Flt1 results in podocyte actin cytoskeleton rearrangements, foot process effacement, and proteinuria [69]. Although the mechanisms by which the dysregulation of sphingolipid metabolism leads to podocyte injury is not well-understood, a variety of disorders highlight the importance of sphingolipids in podocyte health. Many of these disorders are caused by the inability to catabolize certain sphingolipids, also known as sphingolipidoses. An overview of diseases caused by mutations in sphingolipid enzymes is shown in Table 1.

### 4.1. Gaucher Disease

Gaucher disease is an autosomal recessive disorder caused by mutations in the *GBA1* gene which encodes the β-glucosylcerebrosidase protein. β-glucosylcerebrosidase is responsible for the cleavage of glucose from glucosylceramides to produce ceramide. Mutations in *GBA1* result in the impairment in cleaving the glucose of glucosylceramides, and, thus, the accumulation of glucosylceramides occurs [67]. Symptoms of Gaucher disease include spleen and liver malfunction, and skeletal and hematological disorders, as well as an increased risk of developing Parkinson’s disease [70]. The accumulation of glucosylceramides inside of macrophages form “Gaucher cells” which display morphological alterations. Gaucher disease is not commonly associated with nephrotic syndrome; however, cases have been observed. Interestingly, in the instances in which Gaucher disease resulted in nephrotic syndrome, the presence of Gaucher cells in glomeruli was observed [71,72]. Importantly, in one case, treatment with enzyme replacement therapy without the use of therapies for glomerular disease resulted in the complete disappearance of nephrotic symptoms [73]. However, whether the progression of nephrotic syndrome in certain patients with Gaucher disease is due to the accumulation of glucosylceramides in podocytes and whether podocytes can become “Gaucher cells” remain undetermined.

### 4.2. Fabry Disease

Fabry disease is an X-linked disorder caused by mutations in the *GLA* gene which encodes for α-galactosidase. α-galactosidase is responsible for catalyzing the removal of the terminal galactose from globotriaosylceramide (Gb3), a globoside containing three sugar moieties, to generate lactosylceramide. Fabry disease is associated with whole body pain, cardiac impairment, cornea dystrophy, and chronic proteinuria nephropathy, often leading to end-stage renal failure and early death. The kidneys of patients with Fabry’s disease show glomerular hypertrophy with foamy bodies, mesangial thickening, and the formation of zebrabodies in the glomeruli [74]. The deposition of Gb3 in podocytes is age-dependent and progressive [75,76]. Early Gb3 accumulation results in podocyte foot process effacement with further deposition leading to detachment and podocyturia. Interestingly, it seems that the accumulation of lyso-Gb3 induces podocyte stress through the regulation of the uPAR/αvβ3 integrin system and RIPK pathway which drive podocyte injury [77,78]. Treatment with α-galactosidase enzyme replacement is effective in slowing the progression of Fabry disease and the associated renal failure through the increased degradation of Gb3 and Lyso-Gb3 including in podocytes [79,80]. Knockdown of α-galactosidase in immortalized podocytes led to the increased formation of autophagosomes and loss of mTOR kinase activity [81]. However, whether dysregulated autophagy in podocytes caused by Gb3 accumulation is the mechanism of podocyte injury in Fabry’s disease, and whether targeting it is beneficial, is still not fully understood. However, it was recently published that enzyme replacement therapy is not beneficial in reducing glomerular injury in Fabry’s disease and that reducing Gb3 accumulation is not sufficient to prevent podocyte injury [82].

### 4.3. Farber Disease

Farber disease is an autosomal recessive genetic disorder due to mutations in the gene responsible for acid ceramidase (*ASAH1)*. Acid ceramidase is responsible for the removal of fatty acids from ceramides to generate sphingosine. This leads to widespread accumulation of ceramides throughout tissues including the kidneys. Symptoms are most notable in the nervous system, joints, and tissues; however, the liver, heart, and kidneys are often affected as well. Farber disease usually leads to death by the age of 2 years old due to respiratory failure [83]. There is no treatment for Farber disease currently; however, recently, a group has shown that pharmacologically inhibiting acid sphingomyelinase to prevent ceramide accumulation is beneficial in a mouse model of Farber disease. Interestingly, the group recorded elevated levels of blood urea nitrogen (BUN) in their mouse model of Farber disease, which was improved by inhibiting acid sphingomyelinase [84]. Recently, a group has also generated a podocyte-specific *ASAH1* knockout mouse line. These mice displayed severe proteinuria, foot processes effacement, and elevated ceramide levels. Double-knockout mice that lack *SMPD1* (acid sphingomyelinase) displayed decreased glomerular ceramide content and podocyte injury [85]. These studies suggest that acid ceramidase is essential for proper podocyte function. 

### 4.4. Niemann–Pick

Niemann–Pick disease is an autosomal recessive disorder caused by mutations in *SMPD1* causing a deficiency in acid sphingomyelinase and, thus, an accumulation of sphingomyelin. The disease is divided into Type A (infantile) and Type B (visceral). Patients with Type A display severe neurological dysfunction and usually die before the age of 3 years old. Those with Type B do not show neurological dysfunction but exhibit hepatosplomegaly and lung dysfunction. Renal phenotypes are not common, but renal phenotypes including glomerular sclerosis, proteinuria, and the accumulation of lipid droplets in podocytes have been described in some patients [86,87].

### 4.5. Hereditary Inclusion Body Myopathy 2

Hereditary inclusion body myopathy 2 (HIBM) is an autosomal recessive muscular degenerative disorder caused by mutations in the *GNE* gene which encodes the bifunctional enzyme uridine diphospho-N-acetylglucosamine (UDP-GlcNAc) 2-epimerase/N-acetyl-mannosamine (ManNAc) kinase (GNE/MNK). GNE/MNK is responsible for the catalyzation of the first steps of the synthesis of acetylneuraminic acid (Neu5Ac), a component of gangliosides [88]. Interestingly, a mouse model of HIBM, in which the prevalent p.M712T kinase domain mutation was knocked into the endogenous mouse *Gne* through homologous recombination, died within 72 h of birth and displayed severe glomerular disease including proteinuria, podocytopathy, the segmental splitting of the glomerular basement membrane, and the effacement of the podocyte foot processes. Podocytes of these mice displayed a reduced sialyation of podocalyxin, a sialoprotein. The oral administration of the sialic acid precursor N-acetylmannosamine to pregnant and nursing mothers of the knock-in resulted in increased survival and improved renal phenotypes of the offspring [89]. Although the importance of sialyation in podocytes is highlighted in this study, the role of gangliosides to podocyte health were not evaluated. 

### 4.6. Diabetic Kidney Disease

Diabetic kidney disease (DKD) is the lead cause of renal failure in the United States. Podocyte injury is one of the first signs of DKD and drives the progression of renal failure and proteinuria [90]. Sphingolipid accumulation, e.g., of ceramides, sphingosine, sphinganine, and glycosphingolipids, in the plasma of patients with DKD was reported [91,92,93,94,95]. 

In rats, streptozotocin (STZ)-induced diabetes results in an increase in the enzyme activity of neutral ceramidase and sphingosine kinase and the levels of S1P in the glomeruli [96]. Although the origin of S1P was not identified, it would likely have an effect on all cells of the glomerulus due to the paracrine-signaling ability of S1P. Knockout of *SPHK2* in STZ-injected mice is capable of completely preventing albuminuria [78]. Whether this is due to modulating sphingolipid species is not fully understood. Interestingly, it was recently shown that apolipoprotein M, the physiological carrier of S1P, was decreased in the sera of patients with type 2 diabetes. The knockout of apolipoprotein M also exacerbated the progression of glomerular disease in an STZ-injected mouse model, while apolipoprotein M overexpression through adenoviral vector injection reduced diabetic nephropathy phenotypes [97]. 

Interestingly, another group found that STZ-injected mice had reduced glomerular GM3 and sialic acid levels compared to control which were improved following treatment with insulin [98]. Patients with diabetic nephropathy also show reduced glomerular GM3. Treatment of STZ-injected mice with valproic acid, in order to increase GM3, was shown to reduce podocyte injury and improve renal outcomes [99]. 

Our group has shown that sphingomyelin phosphodiesterase acid-like 3b (SMPDL3b) expression is increased in glomeruli from patients with DKD, in glomeruli of db/db mice, and in human podocytes treated with DKD sera [100]. We have previously reported that SMPDL3b impairs insulin receptor isoform B-dependent pro-survival insulin signaling by interfering with the binding of insulin receptor isoforms to caveolin-1 [45]. SMPDL3b displays a homology to acid sphingomyelinase but seems to have a different function as it seems to dephosphorylate C1P rather than catabolize sphingomyelin. SMPDL3b overexpressing human podocytes have reduced levels of C1P similar to what is seen in kidney cortices of db/db mice [101]. Podocyte-specific SMPDL3b deficiency is sufficient to reduce DKD progression, increase C1P levels, and restore insulin receptor signaling in db/db mice. Furthermore, C1P supplementation is sufficient to restore insulin receptor signaling and reduce DKD progression in vivo [45]. Interestingly, targeting acid sphingomyelinase may hold therapeutic benefits in mouse models of diabetic nephropathy as well. It was recently published that podocyte-specific knockdown of acid sphingomyelinase improves podocyte health in a high-fat-diet mouse model by reducing the activation of NLRP3 inflammasome [102].

### 4.7. Puromycin Aminonucleoside (PAN)-Induced Nephropathy

Acute PAN injection in mice and rats induces acute podocyte toxicity and injury, which can eventually lead to glomerulosclerosis, while chronic PAN injection is a model for secondary FSGS. PAN injections in rats was shown to reduce the amount of GD3 and O-acetyl GD3 in a dose-dependent, as well as a time-dependent, manner preceding the development of proteinuria [103]. PAN treatment of human podocytes in vitro also results in a loss of sialic acid and a generation of superoxide anions, which is prevented with sialic acid supplementation, possibly due to the increased superoxide dismutase seen with supplementation [104]. Whether the effect of PAN on podocyte health is due to the reduction in GD3 and O-acetyl GD3 remains undetermined; however, a reduction in gangliosides in the slit diaphragm may reduce the negative charge of the filtration barrier and affect permeability. 

### 4.8. Focal Segmental Glomerulosclerosis

Focal segmental glomerulosclerosis (FSGS) is the leading cause of nephrotic syndrome in adults [105]. We have shown that the number of SMPDL3b-positive podocytes is reduced in post-reperfusion biopsies of patients that developed a recurrence of FSGS [106]. Similarly, in vitro, we showed that human podocytes treated with sera from patients with FSGS have a reduced SMPDL3b expression [100]. These podocytes were also characterized by actin cytoskeleton remodeling and increased apoptosis which was reduced by the overexpression of SMPDL3b or by treatment with rituximab, which binds to SMPDL3b in podocytes [106]. We have also shown that SMPDL3b mediates its effects by interacting with suPAR and affecting aVβ3 activation and, thus, altering RhoA activity [100]. Recently, it was reported that patients with FSGS have lower levels of glomerular GM3, and that GM3 negatively correlates with proteinuria in these patients [107]. Similar results were also reported for patients with minimal change disease. GM3 may have therapeutic potential as increasing GM3 levels through valproic acid reduces proteinuria and podocyte damage in a mouse model of FSGS [108,109]. It seems that the apolipoprotein M and S1P pathway may also be altered in patients with FSGS. It was recently shown by our group that patients with FSGS have a decreased glomerular and plasma apolipoprotein M expression while the SPHK1 and S1PR1-5 expression are increased [110]. Whether apolipoprotein M or S1P signaling play a causal role in FSGS needs to be further explored. 

### 4.9. S1P Lyase

S1P lyase is responsible for the degradation of S1P into hexadecenal and ethanolamine. Interestingly, a group of mutations have been identified in *SGPL1,* the gene responsible for S1P lyase, which cause nephrotic syndrome [111]. The role of S1P lyase has been further explored in mice where S1P lyase deficiency is sufficient to cause podocyte damage and proteinuria [112]. Whether or not the inability to degrade S1P causes podocyte damage due to increased S1P and S1P receptor signaling is unknown, as ceramide and other sphingolipids were also increased in the podocytes due to the inability to degrade all classes of sphingolipids. Podocytes have been shown to express S1P receptor 1–4; however, their function and therapeutic potential in the treatment of glomerular disease have not been explored [113]. 

### 4.10. APOL1 Genetic Alterations

APOL1 genetic risk alleles (G1/G2) have been identified as susceptibility factors for the development of several nephropathies including HIV-associated nephropathy and shown to contribute to podocyte injury [114,115,116]. Multiple mechanisms have been identified in which APOL1 mutants may cause podocyte injury [117,118,119]. Recently, sphingolipid alterations were identified in podocytes that had been transfected with APOL1 genetic variants [120]. The overexpression of APOL1 risk variants led to an enrichment of Gb_3_Cer and DSGb_5_Cer in lipid rafts, while Cer, LacCer, GM_3_, GD_1α_, Gb_5_Cer, and GA1 were decreased. Since lipid rafts are involved in the entry of HIV into cells, the alterations in the sphingolipid composition of lipid rafts may affect the ability of HIV to enter cells [121].

### 4.11. Non-Alcoholic Steatohepatitis 

Non-alcoholic steatohepatitis (NASH) is a risk factor for the development of chronic kidney disease. Recently, it was shown that a mouse model of NASH develops proteinuria, podocyte foot effacement, glomerulosclerosis, elevated BUN, and renal fibrosis. Mice that underwent orthotopic liver transplantation had improved renal outcomes compared to sham surgery mice. Interestingly, the kidneys of the NASH mouse model had increased levels of sphingomyelin [14:0], sphingomyelin [18:1], and ceramide [14:0] [122]. However, whether these sphingolipids alterations contribute to podocyte injury has not been explored. 

### 4.12. Radiation Nephropathy

Radiation therapy for abdominal or paraspinal tumors can lead to radiation nephropathy and podocyte injury. A single dose of radiation increases a variety of ceramide species, decreasing *SMPDL3b* expression, and decreasing S1P levels in podocytes. The overexpression of *SMPDL3b* in podocytes prevents these alterations and prevents cytoskeletal rearrangements, reduces reactive oxygen species generation, and improves double-strand break repair associated with radiation treatment, suggesting a protective effect [123,124,125].

## 5. Concluding Remarks

Sphingolipids are the most diverse class of lipids, and their functional roles vary greatly. Sphingolipids have an important role in the maintenance of the glomerular filtration barrier and in podocyte health. Their importance in podocyte health has been highlighted by the various podocytopathies that occur due to or in association with alterations in the sphingolipid content of podocytes. Although the exact mechanisms as to how alterations in sphingolipid metabolism cause podocyte injury are not fully understood, increasing research suggests that targeting sphingolipids may be beneficial in various glomerular diseases. 

## Figures and Tables

**Table 1:** Overview of primary sphingolipid diseases associated with podocyte damage.

Disease	Genetic Mutation	Sphingolipid Alterations
Gaucher Disease	GBA1 (β-glucosylcerebrosidase)	Glucosylceramide accumulation
Fabry Disease	*GLA* (α-galactosidase)	Globotriaosylceramide accumulation
Farber Disease	*ASAH1* (Acid ceramidase)	Ceramide accumulation
Niemann–Pick	*SMPD1* (Acid sphingomyelinase)	Sphingomyelin accumulation
Hereditary Inclusion Body Myopathy 2	*GLE* (UDP-N-acetylglucosamine 2-epimerase/N-acetylmannosamine kinase)	Reduced sialyation
S1P Lyase Mutation	*SGPL1* (S1P Lyase)	Accumulation of S1P
